# Development of Gated Pinned Avalanche Photodiode Pixels for High-Speed Low-Light Imaging

**DOI:** 10.3390/s16081294

**Published:** 2016-08-15

**Authors:** Tomislav Resetar, Koen De Munck, Luc Haspeslagh, Maarten Rosmeulen, Andreas Süss, Robert Puers, Chris Van Hoof

**Affiliations:** 1 KU Leuven, ESAT, Kasteelpark Arenberg 10, B-3001 Leuven, Belgium; puers@esat.kuleuven.be (R.P.); chris.vanhoof@imec.be (C.V.H.); 2 Imec, Kapeldreef 75, B-3001 Leuven, Belgium; koen.demunck@imec.be (K.D.M.); luc.haspeslagh@imec.be (L.H.); Maarten.Rosmeulen@imec.be (M.R.); andreas.suess@imec.be (A.S.)

**Keywords:** APD, avalanche photodiode, CIS, CMOS, high-speed, image sensor, PAPD, pinned, pixel

## Abstract

This work explores the benefits of linear-mode avalanche photodiodes (APDs) in high-speed CMOS imaging as compared to different approaches present in literature. Analysis of APDs biased below their breakdown voltage employed in single-photon counting mode is also discussed, showing a potentially interesting alternative to existing Geiger-mode APDs. An overview of the recently presented gated pinned avalanche photodiode pixel concept is provided, as well as the first experimental results on a 8 × 16 pixel test array. Full feasibility of the proposed pixel concept is not demonstrated; however, informative data is obtained from the sensor operating under −32 V substrate bias and clearly exhibiting wavelength-dependent gain in frontside illumination. The readout of the chip designed in standard 130 nm CMOS technology shows no dependence on the high-voltage bias. Readout noise level of 15 $\;e^{-}$ rms, full well capacity of 8000$\;e^{-}$, and the conversion gain of 75 µV$/e^{-}$ are extracted from the photon-transfer measurements. The gain characteristics of the avalanche junction are characterized on separate test diodes showing a multiplication factor of 1.6 for red light in frontside illumination.

## 1. Introduction

An image sensor that is suitable for low-light imaging on one hand needs to have good optical properties to collect as many incident photons as possible, and, on the other hand, requires low readout noise levels to bring the signal-to-noise ratio (SNR) close to the Poissonian limit. Suppressing the impact of readout noise can be achieved either by lowering the noise level itself, or by employing a multiplication mechanism in the charge domain. Recent progress in lowering the readout noise of CMOS image sensors (CIS) [1,2,3,4] leaves less and less room for the benefits of electron-multiplying (EM) devices such as EMCCDs, EMCMOS, intensified CCDs (ICCDs) or avalanche photodiodes (APDs), due to the inherent noisiness of the impact-ionization mechanism. Nevertheless, a more thorough comparison of sensors can be made that takes into account their optical properties as well as their speed properties. To compare the low-light performance of different image sensors, one can define a figure of merit (FOM) as the minimum incident photon count *I* that is required to achieve signal-to-noise ratio (SNR) equal to one. This approach is similar to luminance-SNR of 10 (YSNR10) FOM first proposed for mobile-phone cameras [5]. A sensor with lower FOM is therefore desirable for good low-light performance.

(1) $${FOM=I|}_{SNR=1}$$

In order to evaluate the FOM as a function of frame rate (fr), the pixel SNR can be expressed as follows:

(2) $$SNR\left(fr\right)=\frac{QE\;\cdot \;ff\;\cdot \;M\;\cdot \;I}{\sqrt{M^{2}\;\cdot \;F\;\cdot \;\left(QE\;\cdot \;ff\;\cdot \;I+a\;\cdot \;D/fr\right)+\left(1-a\right)\;\cdot \;D/fr+\sigma_{R}^{2}}}$$

taking into account the following parameters: quantum efficiency QE, fill factor ff, multiplication factor *M*, excess noise factor *F*, readout noise $\sigma_{R}$ in electrons rms, and the number of dark electrons per second *D*, where the factor *a* describes the effective fraction of amplified dark current carriers. In this model, the FOM depends on the frame rate only through the dark current. This is especially important in the electron-multiplying devices where—dependent on the position of the EM stage—dark signal carriers can be multiplied together with the photogenerated signal carriers.

**Figure 1:**
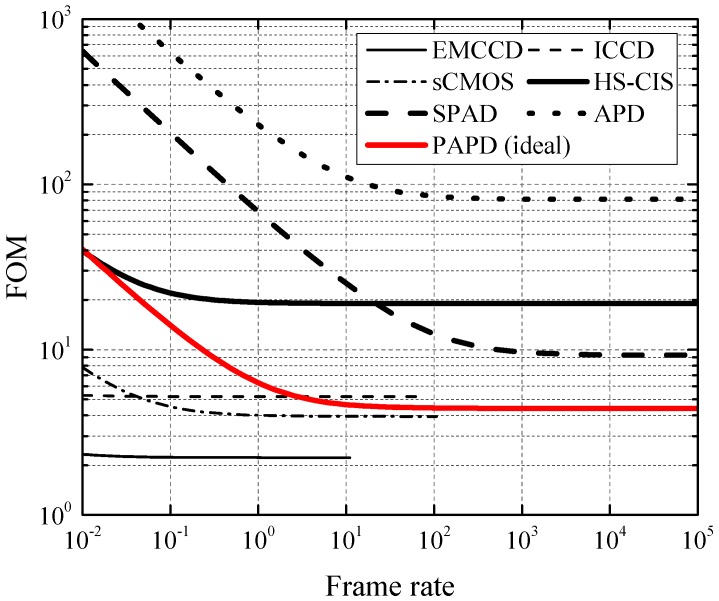
Minimum number of photons incident on a pixel that is required for SNR=1 as a function of frame rate for several typical sensors from literature. Lower figure of merit (FOM) indicates better low-light performance. The red line represents the ideal performance of the gated pinned avalanche photodiode (PAPD) pixel described in this work. APD: avalanche photodiode; EM: electron-multiplying; HS-CIS: high-speed CMOS image sensor; SPAD: single-photon APD; sCMOS: scientific CMOS; ICCD: intensified CCD.

Figure 1 shows FOM plotted with respect to frame rate for several sensors taken from literature, representing the different imaging techniques with their properties listed in Table 1. It can be observed that the lowest FOMs are obtained by sensors that are used in relatively low-speed applications, namely EMCCDs [6], scientific CMOS (sCMOS) [7], and ICCDs [8]. Detailed surveys are available in the literature that cover the trade-offs between those techniques [9]. CCD-based approaches are usually limited in speed by the bandwidth of the output amplifier, or in the case of the EMCCD, the speed of the electron-multiplying stage. Standard four-transistor CIS are not optimized for high-speed operation, and are limited either by the readout speed, by rolling-shutter artifacts, or by image-lag constraints [10]. For these reasons, techniques based on high-speed CIS (HS-CIS) [11], avalanche photodiodes (APDs) [12], or single-photon APDs (SPADs) [13] are used in high-speed imaging. As can be seen from Figure 1, those techniques typically have substantially larger FOMs, leaving room for improvement [14].

High-speed operation of CMOS-based sensors is typically achieved at the cost of both optical properties and readout noise. The thickness of the epitaxial layer needs to be limited in order to avoid slow moving charges generated outside of the depletion region, causing image lag and cross-talk. This effect is especially present in backside-illuminated (BSI) sensors [15]. Limiting the epitaxial layer thickness results in QE reduction for longer wavelengths. HS-CIS are operated in global shutter mode and therefore require more complex pixel architectures than the standard four-transistor pinned photodiode (4-T PPD) pixels. Additional in-pixel storage nodes are employed if correlated double sampling (CDS) capability is desired, giving rise to signal-fidelity problems and sacrificing the pixel photoactive area [11,16]. Due to short frame-times, multiple sampling by the analog-to-digital converters (ADCs) may not be possible, further increasing the total readout noise. These limitations make the charge-multiplying approach using wide-depletion region APDs an interesting area of research, owing to the signal amplification and high speed capability of these devices.

## 2. Prospects of Linear-Mode APDs in High-Speed Low-Light Imaging

Generally, linear-mode APDs and SPADs suffer from poor fill factors when placed in focal-plane arrays. This is due to the presence of guard rings that are needed to prevent premature edge breakdown and additional circuitry that is needed for their operation. Limited literature is available on linear-mode APD CMOS image sensors in contrast to the SPAD-based ones [13,17]. A first attempt has been made in 2001 [18] with poor avalanche noise performance due to the hole-initiated avalanche. More recent work has been presented driven from time-of-flight applications with better noise performance due to the dead space effect [12]. There seems to be two opposite directions in which the problem of the excessive noise factor can be addressed: either very high or very low electric field magnitudes should be targeted. On the one hand, very high electric fields can be formed by increasing the doping concentration of the avalanche junction, thereby forming a very narrow depletion region in which an electron has only a few highly probable opportunities to avalanche. Problems related to this approach stem from the high tunneling currents and poor optical properties due to the very narrow depletion region [19]. On the other hand, lowering the noise factor can be achieved by aiming for lowly-doped junctions that result in low peak electric fields at which the hole-ionization contribution is less pronounced. This is typically expressed by the ratio between hole and electron impact ionization coefficients k=β/α [20]. This approach is successfully applied in EMCCDs, where the charge packet passes through several hundred low-probability ionization opportunities, resulting in a minimum possible noise factor F=2 that is independent of the multiplication factor *M* [21]. In linear mode APDs, such low *k* values are hardly achievable in silicon, since this would require lowly doped junctions that would have to be biased at several hundred volts to achieve the desired gain. If one wants to limit the bias voltage below 100 V, values of *k* below 0.1 seem to be out of reach, resulting in a gain-dependent noise factor [18,22]. This means that—unlike the EMCCD case—the excessive noise factor F is an increasing function of gain M, and therefore an optimal gain exists for SNR improvement for a certain illumination level. In this analysis, the noise factor dependence on gain is described by the following expression [23]:

(3) $$F\left(M\right)=Mk+\left(2-1/M\right)\left(1-k\right).$$

Relative SNR improvement can be estimated as the ratio between the SNR of a device with avalanche multiplication $SNR_{\mathrm{av}}$ and the SNR of the same device without avalanche multiplication $SNR_{\mathrm{nav}}$:

(4) $$\frac{SNR_{\mathrm{av}}}{SNR_{\mathrm{nav}}}=\sqrt{\frac{I+\sigma_{R}^{2}}{FI+\sigma_{R}^{2}/M^{2}}}.$$

**Figure 2:**
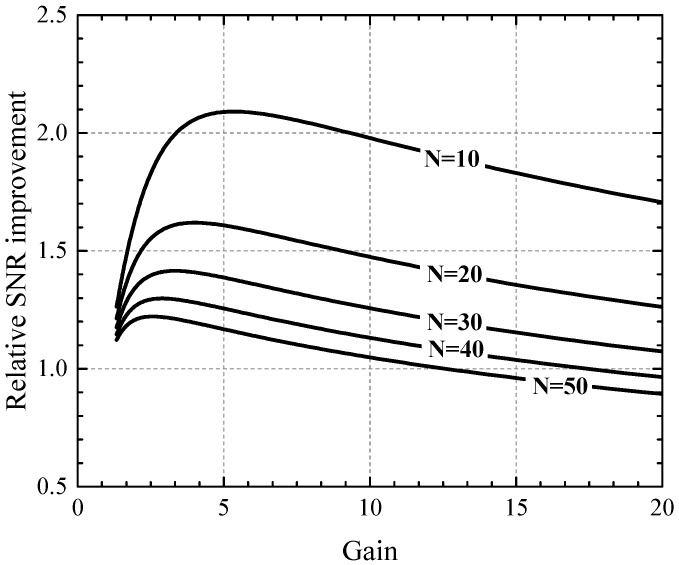
Relative signal-to-noise ratio (SNR) improvement with respect to applied gain for an APD with k=0.1 and readout noise $\sigma_{R}=10\;e^{-}\;\mathrm{rms}$ for different electron counts *N*.

The SNR expression is taken from Equation (2) by omitting the optical factors QE and ff and assuming negligible impact of the dark signal. Relative SNR improvements that can be expected from an APD with k=0.1 and readout noise $\sigma_{R}=10\;e^{-}\;\mathrm{rms}$ from Equation (4) for different electron counts *N* are presented in Figure 2. It can be seen that SNR improvements can be expected for low photon counts, and that, for the presented case, the optimum gain is lower than 5. Increasing the multiplication factor to higher values eventually results in SNR deterioration. It should also be noted that the absolute SNR values at those light levels are already very low, and that single-photon resolution cannot be expected from this approach. The following section therefore discusses further possibilities of employing APDs in single-photon counting.

## 3. Photon Counting with APDs below the Breakdown Voltage

Apart from SPADs that are biased beyond their breakdown voltage ($V_{\mathrm{BD}}$), single-photon detection is also possible with APDs operating below $V_{\mathrm{BD}}$. Very few reports exist in the literature exploring this approach, since the photon detection efficiency is normally assumed to be low [24], and challenges related to bias voltage stability could be present due to the sharp gain characteristics of APDs. Nevertheless, work evaluating this technique for light detection and ranging (LIDAR) applications with APD arrays fabricated in III-V materials report encouraging results [25,26]. More recently, a similar thresholding technique was employed to boost the dynamic range of a standard CMOS image sensor [27]. A short evaluation of the single photon detection probability (PDP) of CMOS APDs with low-noise readout is provided here.

The probability density function (PDF) of APD output carrier count *m*, dependent on the multiplication factor *M*, ratio of hole and electron ionization probability *k*, and input carrier count *N* is derived by McIntyre and Conradi in [28,29]. An approximation of the analytical expression of the PDF that is suitable for numerical calculation is given by:

(5) $$PDF\approx \frac{N}{\sqrt{2\pi m\left(m-N\right)\left[N+k(m-N)\right]}}\times {\left[1-\frac{m/M-N}{m-N}\right]}^{\left(m-N\right)}\times {\left[1+\frac{\left(1-k\right)\left(m-NM\right)/M}{N+k(m-N)}\right]}^{\frac{N+k(m-N)}{1-k}}.$$

**Figure 3:**
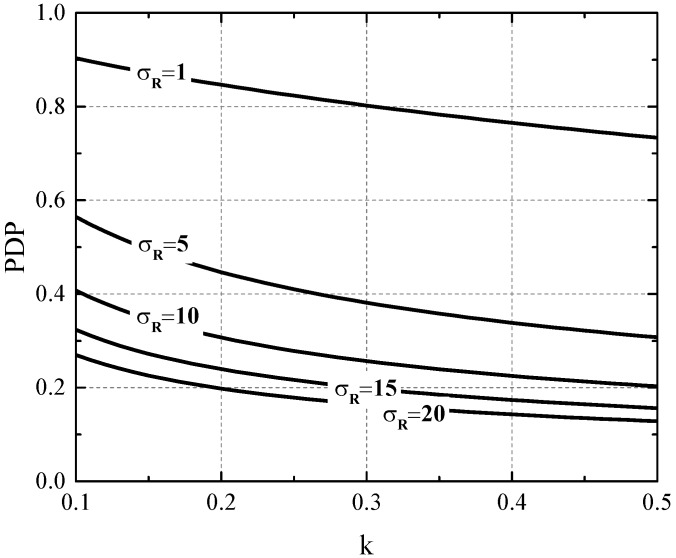
Photon detection probability (PDP) of an APD with M=100 with respect to *k* for different readout noise levels in electrons rms. The detector threshold is set to two times the readout noise rms value.

Assuming a single input carrier N=1 amplified with an average gain of M=100, the single photon detection probability (PDP) can be expressed as:

(6) $$PDP=\int_{N_{\mathrm{th}}}^{\infty }PDFdm$$

where the comparator threshold carrier count is set to two times the the readout noise value $N_{\mathrm{th}}=2\sigma_{R}$ for a 2.3% probability of false detection. Figure 3 represents the PDP with respect to *k* for different readout noise values. It can be seen that an APD with k=0.3, biased below breakdown having a readout noise level of $\sigma_{R}=5\;e^{-}$ can achieve a PDP as high as 35%, which is comparable to the performance of the state-of-the art CMOS SPADs [17]. This approach might therefore be attractive since there is no need for quenching circuitry, and the trade-off between PDP on one hand and dark count rate (DCR) and after pulsing on the other could be mitigated. Moreover, due to lower carrier densities, cross-talk could be improved in shared-well APDs, giving rise to higher fill-factors and smaller pixels [30].

## 4. Backside-Illuminated Gated PAPD Pixel Concept

**Figure 4:**
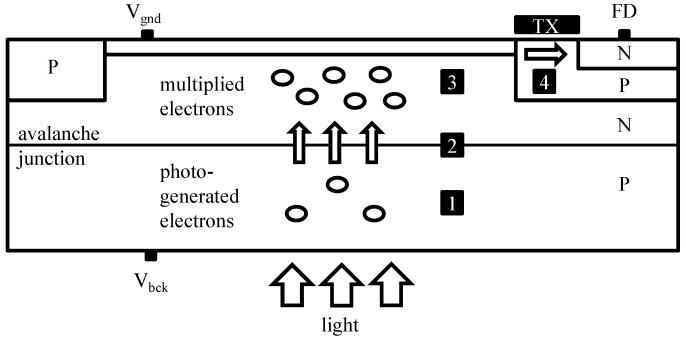
Backside-illuminated (BSI)-gated PAPD pixel concept description in four main phases: 1—light absorption; 2—electron amplification; 3—collection; and 4—transfer. TX: transfer-gate, FD: floating diffusion, P: p-type doped region, N: n-type doped region.

**Figure 5:**
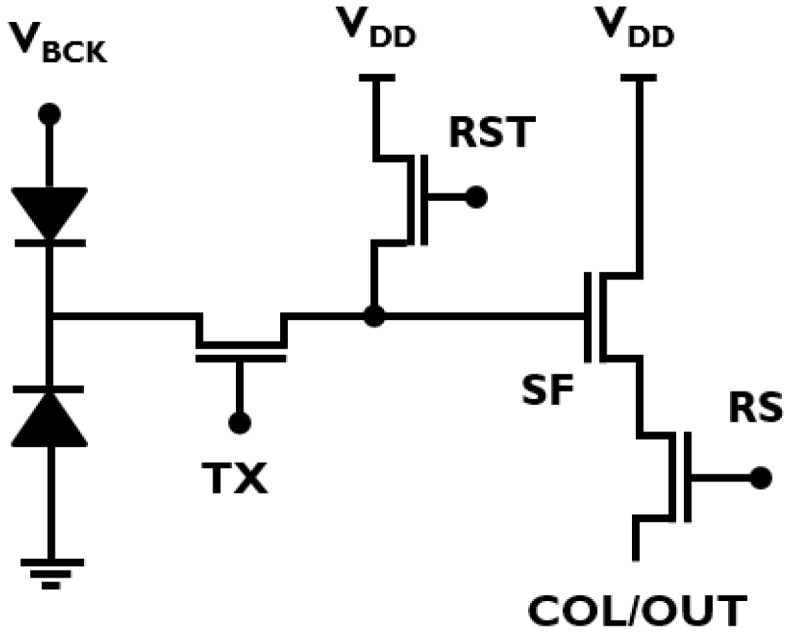
Circuit representation of the gated PAPD pixel.

**Figure 6:**
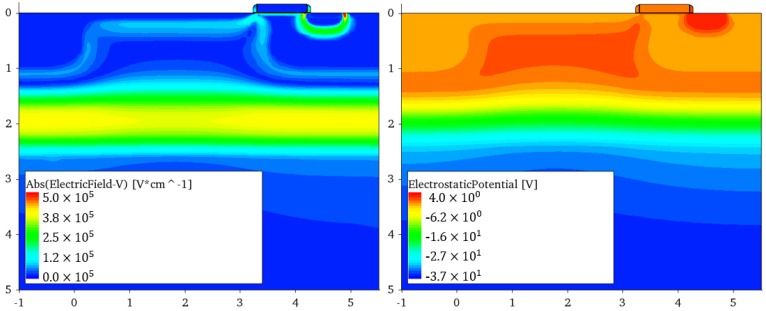
PAPD 2D electric field profile (**left**) and electrostatic potential profile (**right**) from technology computer-aided design (TCAD) at the backside bias voltage $V_{\mathrm{BCK}}=-36$ V.

Recently, a gated pinned APD (PAPD) pixel concept was proposed to explore the possibility of combining standard CIS technology with avalanche signal multiplication, and at the same time providing good optical and speed properties due to full-depletion of the epitaxial layer [14,31]. Figure 4 shows the main principle of operation, with four stages of PAPD operation: 1—BSI light absorption; 2—multiplication; 3—collection; and 4—transfer. As illustrated in Figure 5, besides the high negative voltage $V_{\mathrm{BCK}}$, the pixel can be operated like a standard PPD, since all transistors are isolated from the high voltage. The fill factor problem of conventional APDs and SPADs is mitigated in this approach, since all the pixels in the array share the same avalanche junction and the guard ring preventing premature edge breakdown is implemented only at the edges of the pixel array. The multiplication junction is formed by high-energy boron and phosphorus ion implantations that are added to the standard CIS flow. The doses and energies of the implants were optimized to satisfy two main criteria: firstly, the creation of the high field necessary for impact-ionization at the desired $V_{\mathrm{BCK}}$; and secondly, that both the absorption and the collection regions are fully depleted to avoid shorts and cross-talk between consecutive pixels. The pixel therefore operates at the border of the punch-through breakdown, and special care needs to be taken to maintain a sufficient potential barrier at full depletion. Figure 6 shows a 2D electric field and electrostatic field profile from technology computer-aided design (TCAD) when the pixel is biased at $V_{\mathrm{BCK}}=-36$ V.

**Figure 7:**
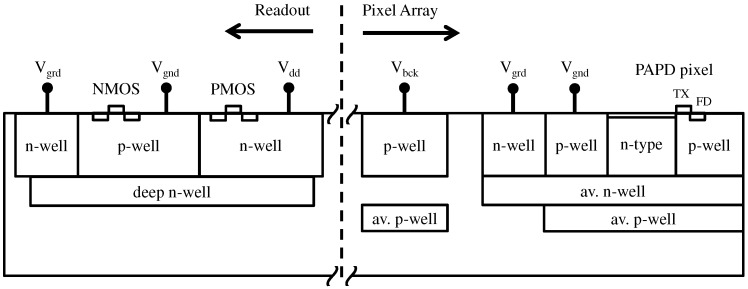
Readout isolation and the pixel array guard-ring implementation.

In order to demonstrate the proof of concept and characterize the basic pixel metrics, a 8 × 32 test pixel array was designed. In addition to the pixel array, a basic readout circuitry was integrated on chip in order to enable row and column addressing and standard four-transistor pixel operation. The readout was designed with 3.3 V CMOS transistors placed in isolated deep wells, as illustrated in Figure 7. It is important that no premature avalanche breakdown happens in the readout part of the chip. From the design perspective, all sharp corners on the outer well edges were avoided in layout, and electrostatic discharge protection structures were modified to withstand the high negative bias. From the technology perspective, with the chosen p-type epitaxial layer with 5.5 µm thickness, the readout avalanche breakdown voltage is −80 V, which imposes the upper limit for the choice of the $V_{\mathrm{BCK}}$ for the pixel operation. Choosing a thicker and more highly resistive epitaxial layer would in principle increase the readout breakdown and enable higher $V_{\mathrm{BCK}}$ values, which would result in increased QE as well as lower *k* values of the avalanche junction, and thus better SNR performance. In this work, a more conservative approach was taken by choosing $V_{\mathrm{BCK}}$ values between −30 and −40 V.

## 5. Experimental Results and Discussion

**Figure 8:**
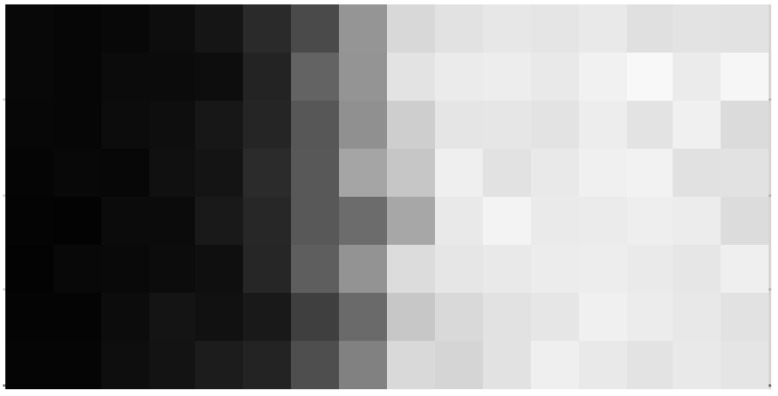
Image taken with a PAPD test array biased at −32 V back bias with left part of the array covered at roughly half full-well capacity.

In this section, preliminary experimental results of the pixel performance are presented. The pixel array consists of 32 rows of floating-diffusion (FD)-shared pixels with 10 µm pitch. It should be pointed out that full characterization was not possible at this time due to an error in the processing of the pixel p-well. As a consequence, the deepest boron implant was not present in the device. For that reason, optimal doping conditions determined in the TCAD analysis were not met, causing shorting of the FD-shared pixels. However, due to wider barriers between pixels that do not share the FD node, sufficient isolation was achieved in binning mode of operation. Therefore, the FD-shared pixels were binned so that the effective pixel size was 10 × 20 µm and the array size was 8 × 16 pixels. Figure 8 shows the first image of a half-covered PAPD test array taken at half of the full-well capacity. No indications of pixel shorts or blooming were observed at operating voltage $V_{\mathrm{BCK}}=-32$ V at these signal levels. The sharpness of the edge should be attributed to the difficulties of projecting a sharp edge onto a small array in our setup. The modulation transfer function (MTF) of PAPD pixels is expected to be high, due to the full depletion of the epitaxial layer. The non-uniformity observed in this image is a combined effect of dark signal non-uniformity (DSNU) and gain non-uniformity.

The PAPD pixel is designed to operate at a fixed backside bias voltage. Increase of the backside voltage results in a sharp increase of the $I_{\mathrm{BCK}}$ current and, consequently, in pixel failure. In agreement with the simulations, only a narrow range of 3V below the punch-through breakdown exists in which the array can be characterized. At lower biases, the pixels are naturally shorted due to the non-depleted n-type layer. The gain properties of the junction therefore cannot be straightforwardly evaluated by sweeping the $V_{\mathrm{BCK}}$. The pixels presented in this work are biased at $V_{\mathrm{BCK}}=-32\;V$, which is several volts below the expected value from TCAD that is required for observation of the predicted optimal gains from Figure 2. The shift in the operating $V_{\mathrm{BCK}}$ is caused by the non-optimal doping conditions due to the mentioned process error. A typical $I_{\mathrm{BCK}}$ current of around 1 nA is measured on an area of $0.15\;{\mathrm{mm}}^{2}$ at the bulk contact of the chip. The pixel dark current of $1\times 10^{5}\;e^{-}$ per second was measured at room temperature, which is two orders of magnitude higher than typically expected in this technology [32]. Due to the reduced boron dose under the shallow trench isolation (STI) region, it is very likely that the depletion region extends to the STI walls. Nevertheless, the dark current is not considered a crucial performance parameter, since this pixel is intended for high-speed operation.

**Figure 9:**
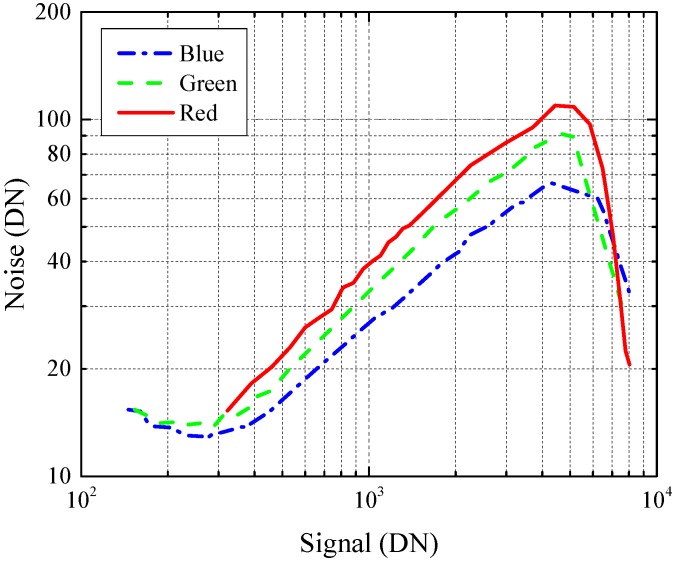
Photon-transfer curves (PTC) for red, blue, and green light, showing the combined impact of avalanche multiplication and noise.

The pixel is primarily being developed for backside illumination where, in principle, the peak backside QE as high as 90% can be achieved with application of proper anti-reflection coatings [33]. However, for proof of concept purposes, no backside processing was applied, and, therefore, only frontside illumination (FSI) characterization was performed. Measured RGB photon-transfer curves (PTCs) of PAPD pixels are presented in Figure 9. The PTCs were acquired by changing the integration time of a pixel under constant illumination. It can be seen that, due to the avalanche multiplication, the PTCs are shifting upwards when illuminated by light of longer wavelength. It should be noted that the observed shift in the PTC is a combined effect of the multiplication factor *M* and the excessive noise factor *F*. For that reason, it is impossible to separate the gain from the noise contribution in the PTC curve. The excessive noise factor can in principle be evaluated with the aid of independent multiplication factor measurements on separate test structures, as discussed at the end of this section. In the present case, due to the limited multiplication factor at this $V_{\mathrm{BCK}}$, the excess noise factor is difficult to estimate with desirable accuracy. Conversion gain of 75 µV$/e^{-}$ was extracted from the blue curve data, which is assumed to have a negligible multiplication factor in FSI. Readout noise of $\sigma_{R}=15\;e^{-}$ rms is observed, which, together with large image lag, indicates that the diode is operating in partially-pinned regime and thus suffering from kTC noise. Full-well capacity of $8000\;e^{-}$ was measured, and is currently limited by the FD node capacitance, which is expected due to the pixel binning. The basic pixel characteristics are summarized in Table 2.

**Figure 10:**
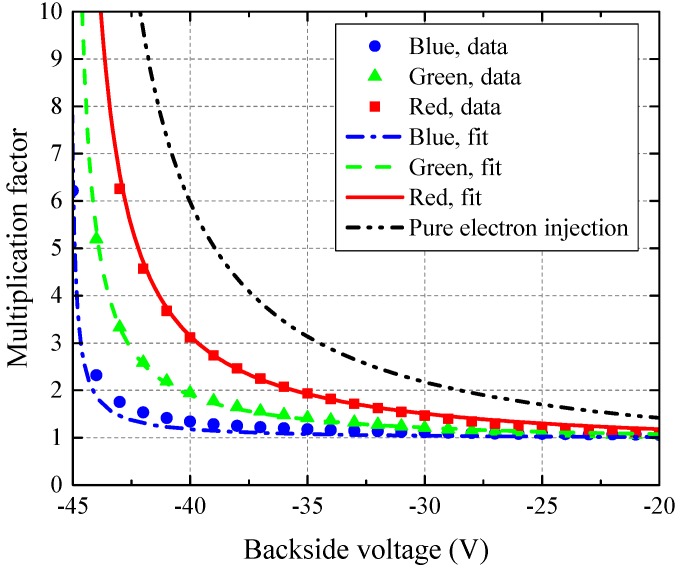
Measurements and analytical fit of avalanche multiplication factor for red, green, and blue light.

Separate test diodes were designed to characterize the multiplication factor of the avalanche junction for a full $V_{\mathrm{BCK}}$ range. A simple model was developed to fit the wavelength dependence of the multiplication factor, which is explained in Appendix A. This model enables the estimation of the pure electron injection multiplication factor which would be observed for blue and green light in BSI. The multiplication factor characteristics are presented in Figure 10 for blue ($\lambda_{b}=470\;\mathrm{nm}$), green ($\lambda_{g}=525\;\mathrm{nm}$), and red ($\lambda_{r}=635\;\mathrm{nm}$) light. It can be seen that biasing the pixel at $V_{\mathrm{BCK}}=-32\;V$ results in a multiplication factor M=1.6 for red light, corresponding to M=2.1 for pure electron injection. This multiplication factor is below the optimal values between 3 and 5, as suggested in Figure 2. According to the presented model, FSI multiplication factors between 1.9 and 2.7 would be desired for red light illumination. It should be noted that in BSI, avalanche gain should depend significantly less on light wavelength than in FSI. For the chosen epitaxial layer thickness of 5.5 µm, carriers that are generated in the 2.5 µm thick region below the avalanche junction all experience the same multiplication factor. Significant wavelength-dependence of the multiplication factor in BSI is therefore expected for wavelengths longer than approximately 580 nm. This effect can be further decreased by placing the avalanche junction closer to the surface, or choosing a thicker epitaxial layer at the expense of higher backside bias needed for the same avalanche gain.

## 6. Conclusions and Outlook

This work shows that, in theory, it is possible to obtain SNR improvements by employing avalanche multiplication in low-light high-speed CMOS imaging. A pixel concept employing avalanche multiplication is proposed which mitigates the need for guardrings and complex circuitry in the pixel array, and is expected to offer good optical and speed properties due to the full depletion of the epitaxial layer. First experimental results of FSI characterization of the proposed pixel are presented. Wavelength-dependent photon-transfer curves due to avalanche multiplication of signal is reported. Nevertheless, because of the unexpected absence of the deepest p-well implant caused by a process error, optimal biasing conditions could not be reached and full depletion of the collection area was not achieved. Further work is therefore needed to obtain devices with higher multiplication factors so that the excess noise factor can be reliably measured in order to evaluate the theoretical predictions of SNR improvement. Aside from proportional signal multiplication, APDs can also be used as photon-counters when biased below their breakdown voltage. Theoretical analysis provided in this paper shows the feasibility of this approach without sacrificing the photon-detection efficiency compared to SPADs. Pixels with substantially higher gains than that presented in this work should therefore be developed by adjusting the implantation doses in order to experimentally demonstrate this approach.

## Figures and Tables

**Table 1:** Sensors from Figure 1 and their properties related to Equation (2).

Sensor	${\mathrm{QE}}_{\max}$	ff	$\sigma_{R}$	*D*	*a*	*M*	*F*	${fr}_{\max}$	Ref.	Comment
EMCCD	0.9	1.0	6.0	$1.0\times 10^{-3}$ *	1.0	1000	2	10	[6]	* deep cooling
ICCD	0.5	1.0	5.4	300	0.0	500	2.6	60	[8]	/
sCMOS	0.6	1.0 *	1.8	0.14	0.0	1.0	1.0	100	[7]	* included in QE
HS-CIS	0.8 *	0.37	5.1	1.0 *	0.0	1.0	1.0	($15\times 10^{3}$)	[11]	* assumed values
SPAD	0.4	0.27	0.17	47	1.0	1.0 *	1.0 *	($16\times 10^{3}$)	[13]	* not applicable
APD	0.23	0.26	26	$5.0\times 10^{4}$	0.0	20	4.5	(800 *)	[12]	* time-of-flight
PAPD *	0.8	1.0	10	10	0.5	3.0	2.1	/	[14]	* ideal values

**Table 2:** Summary of pixel characteristics. FSI: frontside illumination.

Design and Technology		Measured Values	
Pixel count	8×16	Backside bias voltage	−32 V
Pixel dimensions	10 µm × 20 µm (binned)	Dark current @ 25 ${}^{\circ }$C	$1\times 10^{5}$ $e^{-}/s/\mathrm{pixel}$
Fill factor	40% FSI	Multiplication factor @ 635 nm FSI	1.6
Transistors per pixel	4	Readout noise	$15\;e^{-}$ rms
Technology	130 nm 3.3 V CIS	Full-well capacity	8000$\;e^{-}$
		Conversion gain @ 470 nm	75 µV$/e^{-}$

## Appendix Wavelength-Dependent Multiplication Factor Model

The multiplication factor dependence on the applied reverse bias voltage $V_{\mathrm{BCK}}$ of an APD is conventionally expressed as:

(A1) $$M\left(V_{\mathrm{BCK}}\right)={\left[1-{\left(\frac{V_{\mathrm{BCK}}}{V_{\mathrm{BD}}}\right)}^{n}\right]}^{-1}$$

where $V_{\mathrm{BD}}$ is the junction breakdown voltage and *n* is a fitting parameter. Even though this parameter can be used to fit the multiplication data obtained at different wavelengths, here a more physical approach is taken in which a common *n* is found for all wavelengths; thereby, *M* becomes only a property of the multiplication junction. If we consider an APD illuminated from the front side and we assume electrons to be the predominant carrier type in the multiplication process, three distinct regions can be defined. The first region is between the silicon surface and the multiplication junction at depth $x_{2}$; the second region is between the multiplication junction and the end of the epitaxial layer $x_{3}$; and the third region is the highly doped substrate. According to the Beer-Lambert law, the wavelength-dependent portion of the carriers generated in the first region is expressed as:

(A2) $$C_{1}\left(\lambda \right)=\frac{1-\exp\left(-\alpha \left(\lambda \right)x_{2}\right)}{1-\exp\left(-\alpha \left(\lambda \right)x_{3}\right)}$$

where α(λ) is a wavelength-dependent absorption coefficient for silicon. Similarly, the electrons generated in the second region are multiplied with the multiplication factor *M* from Equation (A1):

(A3) $$C_{2}\left(\lambda ,V_{\mathrm{BCK}}\right)=M\left(V_{\mathrm{BCK}}\right)\frac{\exp\left(-\alpha \left(\lambda \right)x_{2}\right)-\exp\left(-\alpha \left(\lambda \right)x_{3}\right)}{1-\exp\left(-\alpha \left(\lambda \right)x_{3}\right)}$$

The electrons generated in the substrate are considered lost due to the rapid recombination process. The final wavelength-dependent multiplication factor can therefore be expressed as a sum of the above contributions:

(A4) $$M\left(\lambda ,V_{\mathrm{BCK}}\right)=C_{1}\left(\lambda \right)+C_{2}\left(\lambda ,V_{\mathrm{BCK}}\right)$$
